# Effectiveness and safety of repetitive transcranial magnetic stimulation on memory disorder in stroke: A protocol for systematic review and meta-analysis

**DOI:** 10.1097/MD.0000000000030933

**Published:** 2022-10-07

**Authors:** Haihua Xie, Dan Xiong, Pan Zhu, Hao Li, Hong Zhang, Jie Tan, Ning Zhao

**Affiliations:** a College of Acupuncture-Moxibustion and Tuina, Hunan University of Chinese Medicine, Changsha, China; b Department of Rehabilitation, The 6th Affiliated Hospital of Shenzhen University Health Science Center, Shenzhen, China.

**Keywords:** memory disorder, meta-analysis, protocol, Repetitive transcranial magnetic stimulation, stroke

## Abstract

Approximately 23% to 55% of patients have memory impairments with a greatly negative effect on daily life 3 months after stroke. Repetitive transcranial magnetic stimulation (rTMS) has been widely used in the rehabilitation of stroke as it is safe, painless, and noninvasive. Moreover, few studies have investigated the effect of rTMS on poststroke memory disorder (PSMD). However, the efficacy of rTMS is not consistent and the optional stimulation frequency is unclear. Therefore, this protocol aims to evaluate the clinical effect and safety of rTMS on PSMD by analyzing results from randomized controlled trials.

**Methods:**

Search strategies will be performed on seven databases: PubMed, EMBASE, CENTRAL, Chinese Biomedical Literature Database (CBM), Chinese National Knowledge Infrastructure (CNKI), Wan Fang, and Technology Periodical Database (VIP). Only randomized controlled trials registered before August 2021 will be included. Additionally, the language will be limited to English or Chinese. For the outcome, we will focus on the Rivermead Behavioral Memory Test. Additionally, the Montreal Cognitive Assessment, Mini-mental State Examination, Modified Barthel Index, and advent events will be included. Two authors will independently select the study, extract data, and assess quality. Moreover, disagreements will be resolved by the third author. STATA 14 and Review Manager 5.4 will be used to perform the analysis. We will evaluate bias risk in accordance with the Cochrane Handbook for Systematic Reviews of Interventions. To assess the quality of evidence, the Grading of Recommendations Assessment, Development, and Evaluation method will be employed.

**Results:**

This study will provide a comprehensive analysis of the current evidence on rTMS for PSMD.

**Conclusion:**

A reliable conclusion regarding whether rTMS is an effective and safe intervention for patients with PSMD and the effect of stimulation frequency and sham stimulation will be provided. This study will provide new insights for TMS in treating PSMD, and offer appropriate treatmentoptions to patients and clinicians.

**PROSPERO registration number:**

CRD42021282439.

## 1. Introduction

Stroke is a global health disease with high rates of morbidity, mortality, and disability.^[1]^ There were 13.7 million patients worldwide diagnosed with stroke in 2016.^[2]^ Moreover, stroke is the third major cause of disability and the second-leading cause of death globally.^[3]^ Poststroke memory disorder (PSMD) are a common consequence of ischemic stroke,^[4]^ and are defined as the inability to remember or recall information or skills.^[5]^ Approximately 23% to 55% of patients 3 months after stroke have memory impairments.^[6]^ An epidemiological study showed that memory impairment accounts for 90% of patients suffering from cognitive impairment.^[7]^ Memory function is one of the highest cognitive functions of humans. Memory disorders are one of the most common cognitive impairments in patients with poststroke cognitive impairment (PSCI) and even the main manifestation in some patients,^[5]^ but not necessarily in all patients with PSCI.^[8]^ It has been demonstrated to have negative impact on the patient’s functional independence and the worst effect on quality of daily life when compared with any other cognitive symptoms.^[9]^ Additionally, van Zelst et al^[10]^ showed that memory is closely linked to speech-motor learning, which indicated that good memory function is beneficial for motor rehabilitation. Therefore, effective memory rehabilitation methods are essential. The current treatments of PSMD include drug therapy, regular rehabilitation therapy, computer-assisted training, virtual reality.^[11–13]^ However, these treatments are minimally effective and insufficient and are plagued by low adherence.^[11,12]^

With the development and progress of technology, a safe, painless, and noninvasive tool, repetitive transcranial magnetic stimulation (rTMS) has been widely applied in the treatment of stroke.^[14]^ After a stroke, the unaffected hemisphere will be more excited and in the dominant position in comparison with the injured hemisphere,^[15]^ which causes a series of complicated pathophysiological events. rTMS utilizes magnetic energy to induce cortical local current, potentiate synaptic plasticity, and change excitability based on the interhemispheric competition model.^[14,16]^ Low-frequency rTMS (≤1 Hz) diminishes unaffected hemisphere excitability, and high-frequency (≥5 Hz) rTMS increases injured hemisphere excitability.^[14]^ In recent years, the possible therapeutic effects of rTMS on memory function have been investigated. Its mechanisms may be related to the regulation of blood flow,^[17]^ mitochondrial energy metabolism,^[18]^ cholinergic neurotransmitters,^[19]^ chemical metabolism,^[20]^ and activation of related brain areas.^[21]^ Preliminary studies have proven that memory performance is improved after rTMS treatment,^[22–24]^ and Rektorova et al^[25]^ did not find a measurable effect of rTMS in memory-related tests after high-frequency rTMS treatment. However, the quality of this literatures varies and the efficacy is controversial which needs further exploration and confirmation. Additionally, the stimulation frequency and treatment of rTMS in the control group was inconsistent for each study. In effective clinical studies, several studies support low-frequency rTMS to enhance memory function,^[22,26]^ while Yin^[24]^ reckons that high-frequency rTMS impacts memory rehabilitation. Some researchers used sham rTMS in the control group, which may have affected the patient’s psychological perception,^[22,23]^ but others did not take any rTMS treatment in the control group.^[24]^ The optimal stimulation frequency and sham stimulation effect are also unclear.

There are many meta-analyses of TMS on post stroke motor dysfunction,^[27]^ cognitive impairment,^[16]^ aphasia,^[28]^ and depression.^[29]^ A meta-analysis of TMS on PSCI has analyzed some outcomes, such as the Montreal Cognitive Assessment (MOCA) and Mini-mental State Examination to explore the efficacy of memory performance,^[16]^ which are usually used to assess global cognition and have some limitations in the assessment of memory function. The Rivermead Behavioral Memory Test (RBMT) is a scale used by professionals to assess memory performance, which is made up of 11 tasks that replicate daily life and are intended to evaluate daily memory function. It is necessary to take it to investigate the exact efficacy of TMS for patients diagnosed with PSMD. Furthermore, it also needs to deeply explore whether stimulation frequency and the sham stimulation will affect its efficacy. To the best of our knowledge, this study is the first meta-analysis to analyze the effect of rTMS on PSMD using RBMT as the primary outcome to supplement the clinical application and clinical practice recommendations of rTMS. The specific purposes of this protocol are as follows:

Aim 1: Is rTMS effective for patients with PSMD?

Aim 2: Is the efficacy different for low-frequency and high-frequency stimulation?

Aim 3: Is there a sham stimulation effect in transcranial magnetic stimulation for memory disorder?

## 2. Methods

Our protocol will adhere to the Preferred Reporting Item for Systematic Review and Meta-analysis Protocols guidelines.^[30]^

### 2.1. Criteria for study selection

#### 2.1.1. Type of studies.

Only randomized controlled trials (RCTs) registered before August 2021 will be included. The language will be limited to English or Chinese.

##### 2.1.1.1. Article exclusion criteria.

Studies that meet one of the following situations will be excluded: duplicated or unextracted data, animal studies, Quasi-experimental studies, conference paper, and editorial material.

#### 2.1.2. Types of participants.

Inclusion criteria: Between 18 and 75 years old. In line with China’s 2015 diagnostic criteria for classifying cerebrovascular diseases or other recognized diagnostic criteria, the first stroke was confirmed by CT or MRI with a course greater than 2 weeks. Memory disorders were diagnosed with related assessment scales and caused by stroke through searching for medical history and interviewing family members that the patients did not manifested memory loss before stroke. Stable vital signs. Awareness. MOCA score < 26.

Exclusion criteria: History of other nervous system diseases. Metal implants, pacemakers, skull defects, or other ailments that could not be treated with rTMS. Mental illness.

#### 2.1.3. Types of interventions.

##### 2.1.3.1. Interventions.

Patients in the trial group receiving active rTMS and RCTs of any stimulation parameters will be included.

##### 2.1.3.2. Comparators.

In the control group, patients did not receive rTMS or received sham rTMS that a stimulation coil was placed on the skull surface but without magnetic stimulation.

##### 2.1.3.3. Combination interventions.

To increase the sample quantity and quality, we will include combination interventions (drug therapy and cognitive rehabilitation training). Only such studies for which the trial and control groups received the same drug therapy or cognitive rehabilitation training will be eligible.

#### 2.1.4. Types of outcome measurements.

##### 2.1.4.1. Primary outcome.

The RBMT will be the primary outcome. It is a professional scale used measure of daily life memory function in stroke,^[22,24]^ and contains story (immediate, delay), picture recognition, route (immediate, delay), messages (immediate, delay), face recognition, orientation and date, appointment, first and second names, and belonging.^[31]^ The maximum score is 24, with scores of 22 to 24 indicating normal memory function, 17 to 21 indicating mild impairment, 10 to 16 indicating moderate impairment, and 0 to 9 indicating severe impairment.

##### 2.1.4.2. Secondary outcomes.

Modified Barthel Index and adverse events will be the secondary outcomes used to respectively assess the safety of TMS and daily living quality. We will also include the MOCA and Mini-mental State Examination to evaluate global cognitive function.

### 2.2. Data search and strategy

Some systematic and comprehensive search strategies will be performed on seven databases: PubMed, EMBASE, CENTRAL, CBM, CNKI, Wan Fang, and VIP.

Generally, our search strategy will be based on the PICO principle containing the keywords “stroke,”^[32]^ “cerebral infarction,” “cerebral hemorrhage,” “transcranial magnetic stimulation,” and “memory.” We will conduct all field searches in the form of Medical Subject Headings and free text on each database. The search strategy of PubMed is supplied in the supplemental content, http://links.lww.com/MD/H541.

### 2.3. Data collection

#### 2.3.1. Study selection.

**Figure 1.**
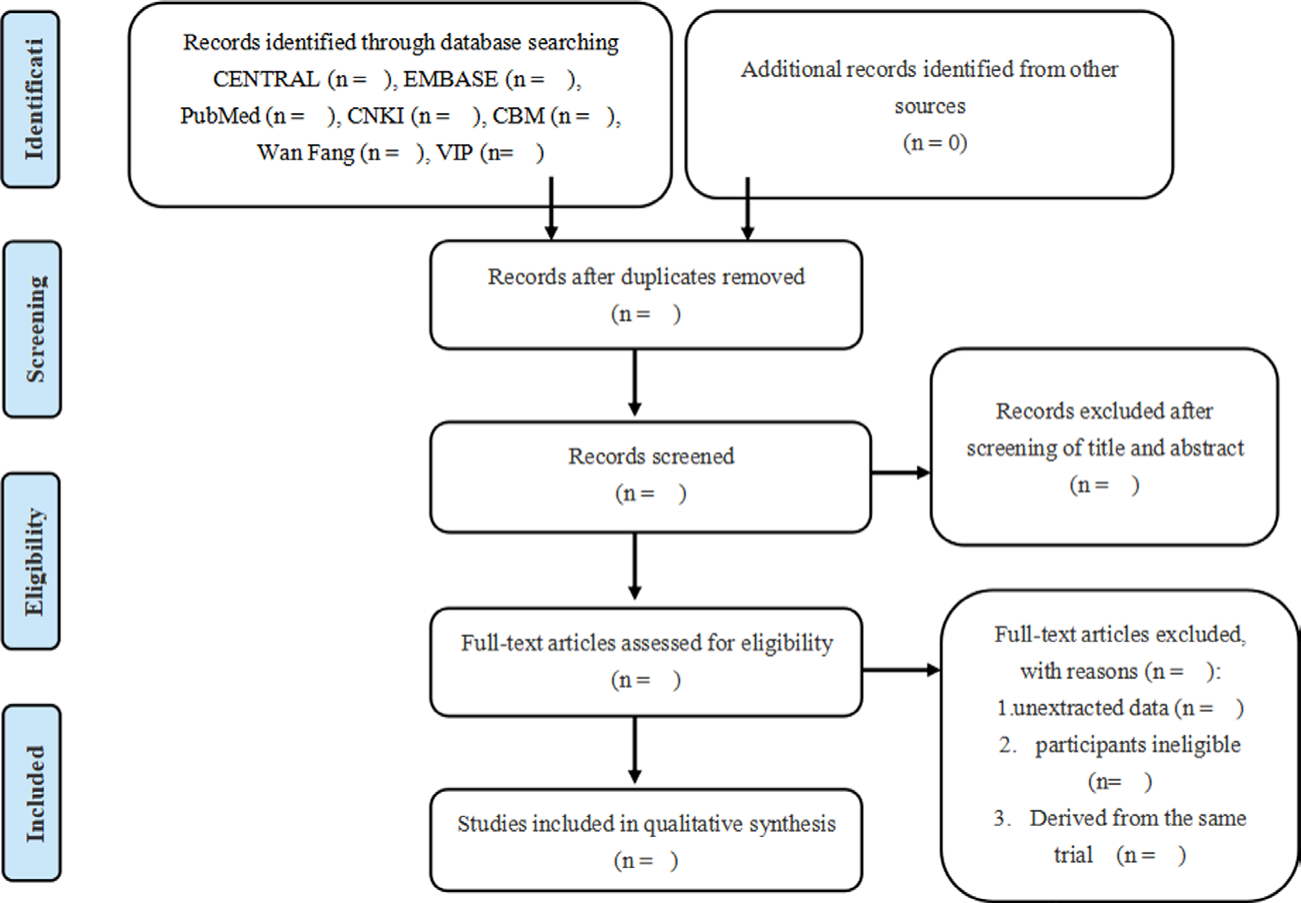
PRISMA flow diagram of study inclusion and exclusion.

All records will be imported into Note Express 3.5.0. Two review authors (DX and PZ) will independently screen abstracts, select the studies in line with the inclusion and exclusion criteria, and crosscheck. Next, two review authors (DX and PZ) will download and independently read the full text of all studies that may meet the requirements for further assessment. The reason for exclusion will be recorded. There will be a third author (HX) to resolve any disagreements. The process is presented in Figure 1.

#### 2.3.2. Data and information extraction.

All available studies that have been assessed by two reviewers will be used to extract data and information. The following information will be independently extracted by two reviewers (DX and PZ) and crosschecked in an advanced-designed Excel file. Data for extraction will include study characteristics (title, the first author, publication year, journalist), trial design (randomization method, blinding, group situation), participants (sample size, mean age, sex ratio, type of stroke, disease course, educational level), intervention (stimulation site, intensity, frequency, single duration, total pulses per session, number of sessions, type of coil, comparison interventions), outcomes (primary outcomes, second outcomes, and quality of outcomes reporting), and adverse events (adverse reactions, adverse time). If there were multiple intervention options in a study, we will strictly follow the principle of chi-squared comparability and select the two datasets with the least heterogeneity.

#### 2.3.3. Solution of missing data.

Generally, we will first try to contact the original authors by e-mail or telephone for missing or incomplete data. If not, they will be excluded in case of bias risk.

### 2.4. Assessment of bias risk

Two reviewers (DX and PZ) will independently assess bias risk of the included studies using the Cochrane risk of bias instrument from the Cochrane Handbook for Systematic Reviews of Interventions.^[33]^ The bias tool embodies seven parts: random sequence generation, allocation concealment, blinding of participants and personnel, blinding of outcome assessment, incomplete outcome data, selective reporting, and other biases. When there are differences of opinion between the two, the third reviewer will make a decision. In addition, the two reviewers will crosscheck for unnecessary errors.

### 2.5. Heterogeneity assessment

Heterogeneity between trials will be tested by the I^2^ statistics. As mentioned in the related articles, there are three possibilities about *I*^2^ statistics: When *I*^2^ = 0, we consider there is no heterogeneity across trials and choose a fixed-effect meta-analysis. If *I*^2^ < 50%, we will also perform a meta-analysis of a random effect model because it represents a low level of heterogeneity. If *I*^2^ ≥ 50%, which denotes a high level of heterogeneity, a random effect model will be selected for more accurate data analysis. Additionally, sensitivity analysis and subgroup analysis will be used to explore the reasons.

### 2.6. Assessment of reporting biases

Reporting biases of all included studies will be assessed by funnel plots on Review Manager 5.4 for the primary outcome measure of RBMT, if there are more than 10 eligible studies. If not, Egger’s test will be taken.

### 2.7. Data synthesis

Data synthesis will be conducted by Review Manager 5.4 and STATA 14 software. Odds ratios and 95% confidence intervals will be analyzed for dichotomous variables. Continuous variables will be expressed as the mean difference and 95% confidence intervals. We will use the fixed-effects model or a random-effects model based on the magnitude of the heterogeneity. A descriptive analysis will be performed when the researches are not suitable for grouping for the excessive heterogeneity.

### 2.8. Subgroup analysis

To further explore the effect of TMS on PSMD, we will conduct the following subgroup analysis: age (more or less than 60), stimulation frequency (low-frequency or high-frequency), sex (male or female), stimulation intensity, length of intervention (more or less 1 month), and treatment in the control group (sham rTMS or without rTMS).

### 2.9. Sensitivity analysis

Sensitivity analysis will be calculated using the leave-one-out method on Review Manager 5.4 to improve the prime outcome’s accuracy and credibility. Some lower sample trials, lower-quality trials, or high heterogeneity trials will be excluded.

### 2.10. Grading the quality of evidence

The Grading of Recommendations Assessment, Development, and Evaluation (http://www.gradeworkinggroup.org/),^[34]^ which contains four levels, high, moderate, low, or very low, to evaluate th**e** quality of evidence, will be applied to estimate the quality of eligible studies.

### 2.11. Patient and public involvement

The type of this article is a protocol for systematic review and meta-analysis based on published or registered research. No patients participated in our study.

## 3. Discussion

Memory disorders are one of the most common impairments in stroke patients with high rates of disability. It can reduce the quality of life of the patient and be devastating for the patient and their family due to its long treatment cycle and poor prognosis. Furthermore, memory function is a critical ingredient of any comprehensive rehabilitation, as intact memory function is a prerequisite for relearning the essence of stroke rehabilitation. However, some existing treatments are ineffective due to poor patient compliance. Thus, we will investigate the effect of rTMS that is applied in the treatment of PSMD for its noninvasive, safe characteristics and its credible theoretical basis in this study.

To our knowledge, this study will be the first meta-analysis to analyze massive data and make a conclusion on whether rTMS has a positive effect and is safe on poststroke memory disorder. Moreover, the results of this study will provide new insights for treating PSMD, supply evidence for better stimulation parameters of rTMS, and offer appropriate treatment options to patients and clinicians.

Nonetheless, there are certainly some limitations to this review. First, as there is no limitation on stimulation parameters in RCTs, heterogeneity may be high. Second, the limitation of language may affect the bias of the report.

## Author contributions

**Conceptualization:** Haihua Xie and Ning Zhao.

**Data curation:** Haihua Xie, Dan Xiong, and Pan Zhu.

**Formal analysis:** Haihua Xie, Dan Xiong, Pan Zhu, Hao Li.

**Methodology:** Haihua Xie, Dan Xiong, Pan Zhu, Hao Li.

**Project administration:** Haihua Xie.

**Supervision:** Hong Zhang, Jie Tan.

**Writing – original draft:** Haihua Xie.

**Writing – review & editing:** Haihua Xie, Ning Zhao, Hong Zhang, Jie Tan.

## Supplementary Material


