# Supplementary material for: Effectiveness and safety of repetitive transcranial magnetic stimulation on memory disorder in stroke: A protocol for systematic review and meta-analysis
Source: Medicine (Baltimore). 2022 Oct 7;101(40):e30933. doi: 10.1097/MD.0000000000030933 (PMC9542838; doi:10.1097/MD.0000000000030933)
Supplement: Supplementary file 1 [file medi-101-e30933-s001.pdf]

Search strategy of PubMed

Number	Terms
#1	stroke[MeSH Terms]
#2	(((((((((((((((((((((((((((Strokes[All Fields]) OR (Cerebrovascular Accident[All Fields])) OR (Cerebrovascular Accidents[All Fields])) OR (CVA (Cerebrovascular Accident[All Fields]))) OR (CVAs (Cerebrovascular Accident[All Fields]))) OR (Cerebrovascular Apoplexy[All Fields])) OR (Apoplexy, Cerebrovascular[All Fields])) OR (Vascular Accident, Brain[All Fields])) OR (Brain Vascular Accident[All Fields])) OR (Brain Vascular Accidents[All Fields])) OR (Vascular Accidents, Brain[All Fields])) OR (Cerebrovascular Stroke[All Fields])) OR (Cerebrovascular Strokes[All Fields])) OR (Stroke, Cerebrovascular[All Fields])) OR (Strokes, Cerebrovascular[All Fields])) OR (Apoplexy[All Fields])) OR (Cerebral Stroke[All Fields])) OR (Cerebral Strokes[All Fields])) OR (Stroke, Cerebral[All Fields])) OR (Strokes, Cerebral[All Fields])) OR (Stroke, Acute[All Fields])) OR (Acute Stroke[All Fields])) OR (Acute Strokes[All Fields])) OR (Strokes, Acute[All Fields])) OR (Cerebrovascular Accident, Acute[All Fields])) OR (Acute Cerebrovascular Accident[All Fields])) OR (Acute Cerebrovascular Accidents[All Fields])) OR (Cerebrovascular Accidents, Acute[All Fields])
#3	Cerebral Infarction[MeSH Terms]
#4	((((((((((((((((((((((((Cerebral Infarctions[All Fields]) OR (Infarctions, Cerebral[All Fields])) OR (Infarction, Cerebral[All Fields])) OR (Cerebral Infarct[All Fields])) OR (Cerebral Infarcts[All Fields])) OR (Infarct, Cerebral[All Fields])) OR (Infarcts, Cerebral[All Fields])) OR (Cerebral Infarction, Left Hemisphere[All Fields])) OR (Left Hemisphere, Infarction, Cerebral[All Fields])) OR (Infarction, Left Hemisphere, Cerebral[All Fields])) OR (Left Hemisphere, Cerebral Infarction[All Fields])) OR (Cerebral, Left Hemisphere, Infarction[All Fields])) OR (Infarction, Cerebral,

	Left Hemisphere[All Fields])) OR (Subcortical Infarction[All Fields])) OR (Infarction, Subcortical[All Fields])) OR (Infarctions, Subcortical[All Fields])) OR (Subcortical Infarctions[All Fields])) OR (Posterior Choroidal Artery Infarction[All Fields])) OR (Anterior Choroidal Artery Infarction[All Fields])) OR (Cerebral Infarction, Right Hemisphere[All Fields])) OR (Right Hemisphere, Cerebral Infarction[All Fields])) OR (Infarction, Right Hemisphere, Cerebral[All Fields])) OR (Right Hemisphere, Infarction, Cerebral[All Fields])) OR (Cerebral, Right Hemisphere, Infarction[All Fields])) OR (Infarction, Cerebral, Right Hemisphere[All Fields])
#5	Cerebral Hemorrhage[MeSH Terms]
#6	((((((((((((((((((((((Hemorrhage, Cerebrum[All Fields]) OR (Cerebrum Hemorrhage[All Fields])) OR (Cerebrum Hemorrhages[All Fields])) OR (Hemorrhages, Cerebrum[All Fields])) OR (Cerebral Parenchymal Hemorrhage[All Fields])) OR (Cerebral Parenchymal Hemorrhages[All Fields])) OR (Hemorrhage, Cerebral Parenchymal[All Fields])) OR (Hemorrhages, Cerebral Parenchymal[All Fields])) OR (Parenchymal Hemorrhage, Cerebral[All Fields])) OR (Parenchymal Hemorrhages, Cerebral[All Fields])) OR (Intracerebral Hemorrhage[All Fields])) OR (Hemorrhage, Intracerebral[All Fields])) OR (Hemorrhages, Intracerebral[All Fields])) OR (Intracerebral Hemorrhages[All Fields])) OR (Hemorrhage, Cerebral[All Fields])) OR (Cerebral Hemorrhages[All Fields])) OR (Hemorrhages, Cerebral[All Fields])) OR (Brain Hemorrhage, Cerebral[All Fields])) OR (Brain Hemorrhages, Cerebral[All Fields])) OR (Cerebral Brain Hemorrhage[All Fields])) OR (Cerebral Brain Hemorrhages[All Fields])) OR (Hemorrhage, Cerebral Brain[All Fields])) OR (Hemorrhages, Cerebral Brain[All Fields])
#7	#1 OR #2 OR #3 OR #4 OR #5 OR #6
#8	Transcranial Magnetic Stimulation[MeSH Terms]
#9	(((((((Magnetic Stimulation, Transcranial[All Fields]) OR (Magnetic Stimulations, Transcranial[All Fields])) OR (Stimulation, Transcranial

	Magnetic[All Fields])) OR (Stimulations, Transcranial Magnetic[All Fields])) OR (Transcranial Magnetic Stimulations[All Fields])) OR (Transcranial Magnetic Stimulation, Single Pulse[All Fields])) OR (Transcranial Magnetic Stimulation, Paired Pulse[All Fields])) OR (Transcranial Magnetic Stimulation, Repetitive[All Fields])
#10	#8 OR #9
#11	Memory[MeSH Terms]
#12	memory[All Fields]
#13	#11 OR #12
#14	#7 AND #10 AND #13
